# The prevalence of polypharmacy and hyper-polypharmacy among middle-aged vs*.* older patients in Saudi Arabia: a cross-sectional study

**DOI:** 10.3389/fphar.2024.1357171

**Published:** 2024-06-12

**Authors:** Aymen A. Alqurain, Murtada Albaharnah, Samanah Al Zayer, Luma Ameer, Sherihan Ghosn, Samaher Al-Shaibi, Marwa Algoraini, Amal Aldhafeeri, Danah A. Alyusuf, Afnan Alshnbari, Nida Alsaffar, Jenan Al-Matouq, Mohammed Al Khamees, Bader AlAlwan, Fadhel A. Alomar

**Affiliations:** ^1^ Department of Pharmacy, Mohammed Al-Mana College for Medical Sciences, Dammam, Saudi Arabia; ^2^ Foundation Year Department, Mohammed Al-Mana College for Medical Sciences, Dammam, Saudi Arabia; ^3^ Pharmaceutical Service Department, Al Mana General Hospital, Khobar, Saudi Arabia; ^4^ Pharmaceutical Service Department, Qatif General Hospital, Al Qaţīf, Saudi Arabia; ^5^ Department of Medical Science, Mohammed Al-Mana College for Medical Science, Dammam, Saudi Arabia; ^6^ Clinical Laboratory Department, King Fahad Hospital in Hoffuf, Al Hufūf, Saudi Arabia; ^7^ Department of Pharmacology, College of Clinical Pharmacy, Imam Abdulrahman Bin Faisal University, Dammam, Saudi Arabia

**Keywords:** older patients, middle-aged patients, medical care unit, polypharmacy (MeSH), hyper-polypharmacy

## Abstract

**Introduction:**

Polypharmacy, the use of multiple medications, is a growing concern among middle-aged and older patients, posing potential risks and challenges in healthcare management.

**Aim:**

This study aimed to identify the prevalence of polypharmacy and hyper-polypharmacy among populations of middle-aged vs*.* older patients and identify its associated common comorbidities and prescribed medications in Qatif Central Hospital (QCH), Saudi Arabia.

**Methods:**

Patients aged 40 years or older who presented to an outpatient medical care clinic at QCH, Saudi Arabia, between 1 January and 31 December 2021 were included, and their comorbidities, prescribed medications, and recent clinical laboratory test results were collected. The Charlson comorbidity index (CCI) score was calculated to predict the risk of mortality. Logistic regression was used to compute the association between the prevalence of polypharmacy and patient characteristics. The results were presented as odds ratios (ORs) and 95% confidence intervals (95% CIs).

**Results:**

A total of 14,081 patients were included; 31% of the cohort comprised older patients, and 66% of the cohort was identified with polypharmacy. The majority of the polymedicated patients were presented to an internal medicine care unit (34%). The prevalence of polypharmacy was positively associated with CCI (OR = 3.4, 95% CI 3.3–3.6), having a disease related to the musculoskeletal system (MSD) (OR = 4.2, 95% CI 3.8–4.7), and alimentary tract and metabolism (ATM) (OR = 3.8, 95% CI 3.4–4.2). Conversely, the prevalence of polypharmacy was negatively associated with age (OR = 0.9, 95% CI 0.89–0.91) and patients with cardiovascular diseases (OR = 0.6, 95% CI 0.5–0.7).

**Conclusion:**

Polypharmacy is still an ongoing concern. Patients, particularly those with diseases related to MSD or ATM, should be considered for reviewing prescriptions by pharmacists to reduce the risk of adverse drug reactions and future consequences of polypharmacy.

## 1 Introduction

Globally, polypharmacy is a major medication safety concern (Alwhaibi et al., 2020). Polypharmacy is commonly known as the concurrent intake of five or more medications (Gnjidic et al., 2012). Hyper-polypharmacy, which involves the prescription of ten or more medications, notably increases the risk of hospitalizations, increases healthcare expenses, and mortality (Mekonnen et al., 2022). It has been estimated that the global prevalence of polypharmacy among older patients accounts for 38%–91%. In the United Kingdom, there were large increases in the number of patients with polypharmacy due to an increase in the use of drugs from 11.4% to 20.8% and increases in the number of patients with hyper-polypharmacy from 1.7% to 5.8% (Guthrie et al., 2015). In Saudi Arabia, recent research reported that the prevalence of polypharmacy among patients who attended outpatient clinics at a tertiary teaching hospital was 46.5%, which was higher than the value reported in Australia, which was 36.1% (Page et al., 2019; Balkhi et al., 2021).

Polypharmacy is linked to an increased risk of harmful effects associated with medication usage (Alsuwaidan et al., 2019b). These harmful effects include nonadherence to drugs, medication errors, drug–drug interaction (DDI) incidents, and adverse drug reactions (ADRs). Polypharmacy can also lead to inappropriate or irrational medication use, which will have a significant negative impact on healthcare resources and costs, leading to a large economic burden (Dagli and Sharma, 2014). Accordingly, the World Health Organization (WHO) has asked countries and the concerned authorities to prioritize medication safety in polypharmacy, take early action, and reduce avoidable medication-related harm (Johansson et al., 2016).

The previous literature had reported several reasons for the occurrence of polypharmacy, including multiple comorbidities and the promotion of consistent treatment of several chronic morbidities by guidelines that recommend multiple drugs therapy (Guthrie et al., 2015). Van Dam et al. reported that polypharmacy and chronic comorbidity could contribute to frailty, increasing the risk of DDIs and ADRs (van Dam et al., 2022). Previous studies reported that polypharmacy was more common in patients with hypertension, hyperlipidemia, and gastric ulcer, whereas medications related to cardiovascular system, gastritis, and hypnotics were the most medication classes reported in patients with polypharmacy (Mizokami et al., 2012).

Evidence-based strategies have been developed and introduced to limit the harmful effects of polypharmacy and promote the optimal use of multiple medications. However, structured management programs and supporting policies are still limited in many countries (Johansson et al., 2016). These strategies emphasize that a comprehensive consideration in the clinical context is needed rather than a simple approach with the number of medications used, which is essential to develop rational policies for improving polypharmacy use (Baruth et al., 2020).

To the best of our knowledge, most studies conducted on polypharmacy in Saudi Arabia have only targeted certain vulnerable patient groups, such as the elderly or those with diabetes (Salih et al., 2013; Alwhaibi et al., 2018a). Nevertheless, the information about the prevalence of polypharmacy or hyper-polypharmacy among younger or middle-aged patients is limited. The objectives of this study are to determine the difference in the prevalence of polypharmacy and hyper-polypharmacy among middle-aged vs*.* older patients who attended a medical care unit, the most commonly reported comorbidities and prescribed medications, and the factors associated with polypharmacy among this cohort of patients.

## 2 Methods

### 2.1 Study population

A retrospective, cross-sectional, descriptive study was conducted to best explain the study objectives as the cross-sectional design is an approach to determine the prevalence of polypharmacy. Data were collected from the electronic medical records of patients who presented to Qatif Central Hospital (QCH), the largest public hospital in the eastern region of Saudi Arabia serving Al-Qatif city, between 1 January and 31 December 2021. Patients aged 40 years or older who attended an outpatient medical care clinic were included in this study. Patients under 40 years of age, who attended surgical, dental, or gynecological/obstetric care units or were admitted to the hospital, were excluded from this study. For patients with multiple visits during the data collection period, only the first reported visit was considered for the purpose of this study.

### 2.2 Data collection, measures, and definitions

Patients’ demographic data and comorbidities were collected from the medical electronic record; the prescribed and dispensed medications were recorded from the pharmacy electronic records, and the medical laboratory test results were collected from the laboratory electronic records.

Comorbidities were identified as reported in the medical record and by applying the Rx-Risk comorbidity index on the prescribed medication list and were then coded as per the International Classification of Disease, 10th revision, 2016 (ICD-10) (Lu et al., 2011; World Health Organisation, 2016). In this study, it was difficult to assess the level of frailty in the included patient due to the retrospective nature of the study. Thus, the Charlson comorbidity index (CCI) score was calculated as a predictor of the 1-year mortality risk and as an approach to evaluate the patient’s frailty level (Charlson et al., 1987; Al-Qurain et al., 2020b). Creatinine clearance (CrCl) was calculated using the Cockcroft–Gault equation (Cockcroft and Gault, 1976).

The prescribed and dispensed medications, including long-term and short-term medications prescribed as needed and supplements, were collected and then coded as per the Anatomical Therapeutic Chemical (ATC) classification system (World Health Organization, 2000). Medications were reported as the first-level or the second-level order of the ATC system throughout the study. Long-term medicines were defined as those that did not have a defined duration of use. From this list, the total number of prescribed medications (NPMs) was counted. Falls risk increasing drugs (FRIDs) and orthostatic hypotension contributing drugs (ODs) were identified and classified according to Milos et al. (2014) and Al-Qurain et al. (2020a).

The included patients were classified based on their age into middle-aged patients (<65 years) or older patients (≥65 years). Polypharmacy was defined by the concurrent intake of five to nine medications, whereas hyper-polypharmacy was defined by the concurrent intake of 10 or more medications (Gnjidic et al., 2012). Based on the polypharmacy levels, patients were classified as non-polypharmacy (prescription of <5 medications), polypharmacy (prescription of 5–9 medications), or hyper-polypharmacy (prescription of ≥10 medications). Finally, to help in assessing the trend pattern, patients were classified based on their age into six different groups (40–49, 50–59, 60–69, 70–79, 80–89, and 90 years or older).

### 2.3 Ethical consideration

This study was approved by the Institutional Review Board (IRB) at Mohammed Al-Mana College for Medical Sciences (SR/RP/79) and the IRB at QCH (QCH-SREC019/2022).

### 2.4 Statistical analysis

Demographic variables, comorbidities, and medication use were reported using mean and standard deviation (SD) for continuous parametric variables, median/interquartile range (IQR) for continuous non-parametric variables, and the number/frequency for binary variables. For comparisons of continuous variables, Student’s t-test for the parametric or the Mann–Whitney *U* test for the non-parametric data was used, whereas the chi-squared test was used to compare the frequency of categorical variables between the groups. The analysis of variance (ANOVA) test was used to identify any significant change in different age groups. Binary logistic regression was performed to compute unadjusted and adjusted odds ratios (ORs) and 95% confidence interval (95% CI) to describe the association between polypharmacy and the patients’ demographic variables, comorbidities, and prescribed medications among the cohort, middle-aged patients, or older patients. The binary logistic regression was adjusted with age, gender, CCI, FRIDs, OD, and CrCl. Covariates were included in the models if they reached a level of statistical significance at *p* < 0.05 in univariate analysis. Multicollinearity was tested with the variance inflation factor. Statistical analysis was performed using the SPSS statistical package version 26 (SPSS, Inc., Chicago, IL), and *p* ≤ 0.05 was considered statistically significant.

## 3 Results

A total of 14,081 patients were included in this study, of which 54% (n = 7651) were female individuals, and the prevalence of those who were older patients was 31% (Table 1). Table 1 shows that middle-aged patients were heavier (76.1 kg vs*.* 73.1 kg, *p* < 0.001), had a higher CrCl value (93.8 mL/min vs*.* 60.3 mL/min, *p* < 0.001), and had a lower median CCI score (2 vs*.* 5, *p* < 0.001) compared to older patients. Older patients were prescribed more medications (8 vs*.* 6, *p* < 0.001), more OD medications (1 vs*.* 0, *p* < 0.001), and less average FRID medications (0.25 vs*.* 0.3, *p* < 0.001) compared to middle-aged patients (Table 1). Table 1 also shows that most of the cohort attended an internal medicine (31%), a rheumatology (16%), a cardiovascular (9%), a neurology (9%), and an endocrinology (7%) care unit. The majority of the middle-aged and older patients attended an internal medicine unit (37% vs*.* 28%, *p* < 0.001). Compared to older patients, middle-aged patients attended a rheumatology unit more (18% vs*.* 12%, *p* < 0.001) and a cardiovascular unit less (7% vs*.* 13%, *p* < 0.001).

**TABLE 1:** Characteristics of the patients included in the study classified based on their age group. BMI, body mass index; CCI, Charlson comorbidity index; NPM, number of prescribed medications; FRIDs, falls risk increasing drugs; OD, orthostatic hypotension-contributing drugs; CrCl, creatinine clearance; HB, hemoglobin.

Characteristic	Cohort	Middle-aged adults		Older adults	*p*-value
	*n* = 14,081	*n* = 9,722		*n* = 4,359	
Gender (female), (n %)	7,651 (54)	5,320 (55)		2,331 (54)	0.2
Body weight (kg), mean (SD)	75.2 (18.1)	76.1 (18.6)		73.1 (16.6)	<0.001
BMI (Kg/m^2^), mean (SD)	31.8 (15)	32.2 (15.3)		30.8 (14.1)	<0.001
CCI, median (IQR)	3 (1–7)	2 (0–5)		5 (3–8)	<0.001
NPM, median (IQR)	6 (2–15)	6 (2–15)		8 (2–15)	<0.001
FRID, median (IQR)	0 (0–1)	0 (0–1)		0 (0–1)	<0.001
FRID, mean (SD)	0.3 (0.7)	0.33 (0.7)		0.25 (0.6)	<0.001
OD, median (IQR)	1 (0–3)	0 (0–2)		1 (0–4)	<0.001
CrCl (mL/min), mean (SD)	83.4 (48.9)	93.8 (49.8)		60.3 (38)	<0.001
HB (g/dL), mean (SD)	11.7 (2.3)	11.7 (2.3)		11.7 (2.2)	0.6
Care unit specialty					
Internal medicine, (n %)	4,355 (31%)	2,733 (28%)		1,622 (37%)	<0.001
Rheumatology, n (%)	2,301 (16)	1,779 (18)		522 (12)	<0.001
Cardiology, n (%)	1,282 (9)	713 (7)		569 (13)	<0.001
Neurology, n (%)	1,235 (9)	921 (10)		314 (7)	<0.001
Endocrinology, n (%)	1,037 (7)	765 (8)		272 (6)	<0.001
Urology, n (%)	781 (6)	543 (6)		238 (6)	<0.001
Gastroenterology, n (%)	697 (5)	547 (6)		150 (3)	<0.001
Hematology, n (%)	596 (4)	521 (5)		75 (2)	<0.001
Psychiatric, n (%)	618 (4)	449 (5)		169 (4)	<0.001
Pulmonary, n (%)	574 (4)	371 (4)		203 (5)	<0.001
Infectious disease, n (%)	385 (3)	264 (3)		121 (3)	<0.001
Nephrology, n (%)	219 (2)	116 (1)		103 (2)	<0.001

**FIGURE 1:**
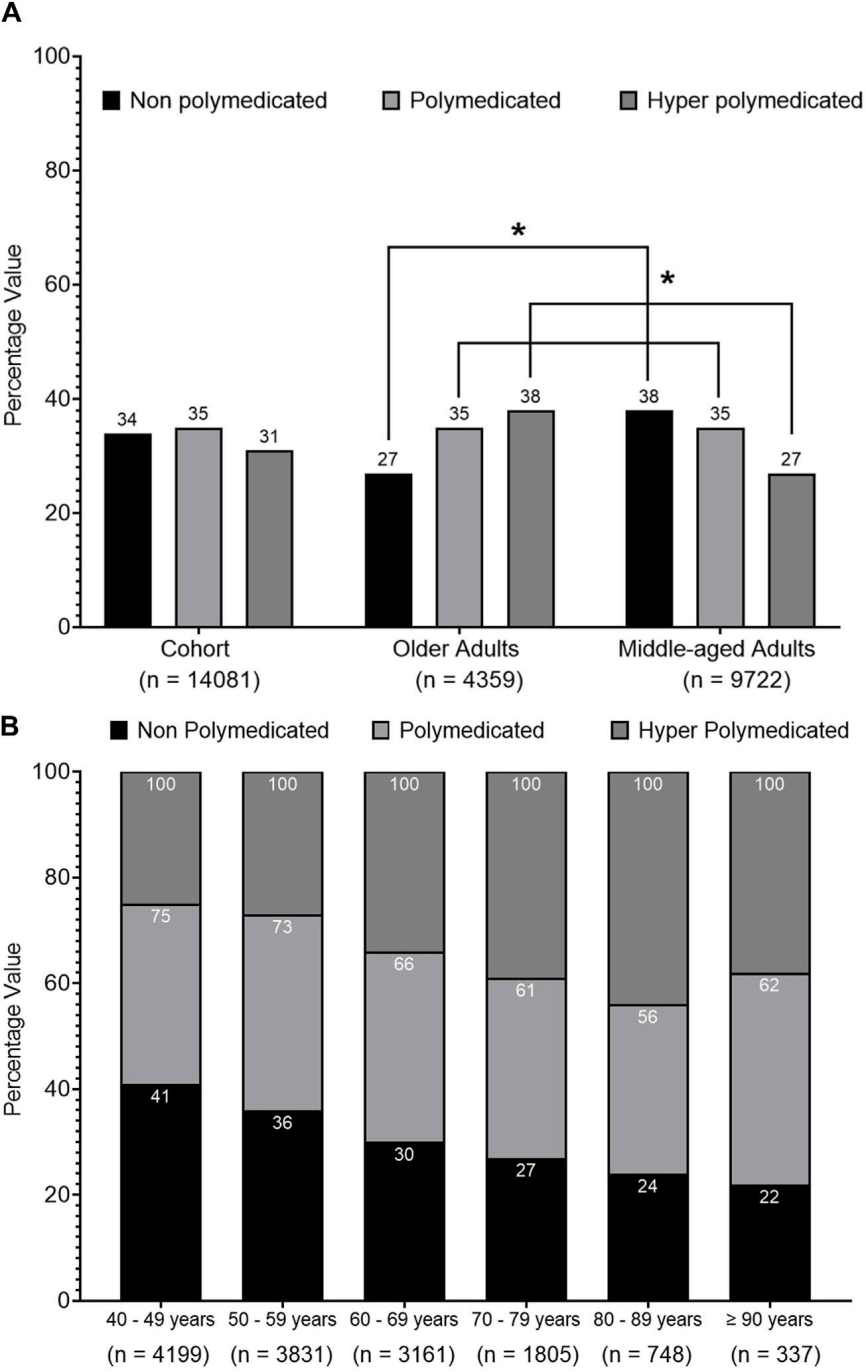
Prevalence of polypharmacy among the cohort. **(A)** Univariate analysis of the prevalence of polypharmacy among the cohort classified into middle-aged and older patients. **(B)** ANOVA analysis of polypharmacy prevalence trend among the cohort classified into different age groups. Data are presented as a percentage value of the referenced group. * = *p* < 0.001.

Figure 1 shows that 66% of the cohort was exposed to polypharmacy, 35% was identified as polymedicated, and 31% was identified as hyper-polymedicated. The prevalence of polypharmacy was higher among older patients compared to middle-aged patients (73% vs*.* 62%, *p* < 0.001). Figure 1 also presents that most of the older patients were identified as hyper-polymedicated (38% vs*.* 27%, *p* < 0.001), whereas the minority were non-polymedicated (27% vs*.* 38%, *p* < 0.001) compared to middle-aged patients. Figure 1A shows an inverse trend between older vs*.* middle-aged patients in terms of being non-polymedicated, polymedicated, or hyper-polymedicated. Figure 1B shows an increasing prevalence trend of polypharmacy and hyper-polypharmacy among each age group with increasing age.

**FIGURE 2:**
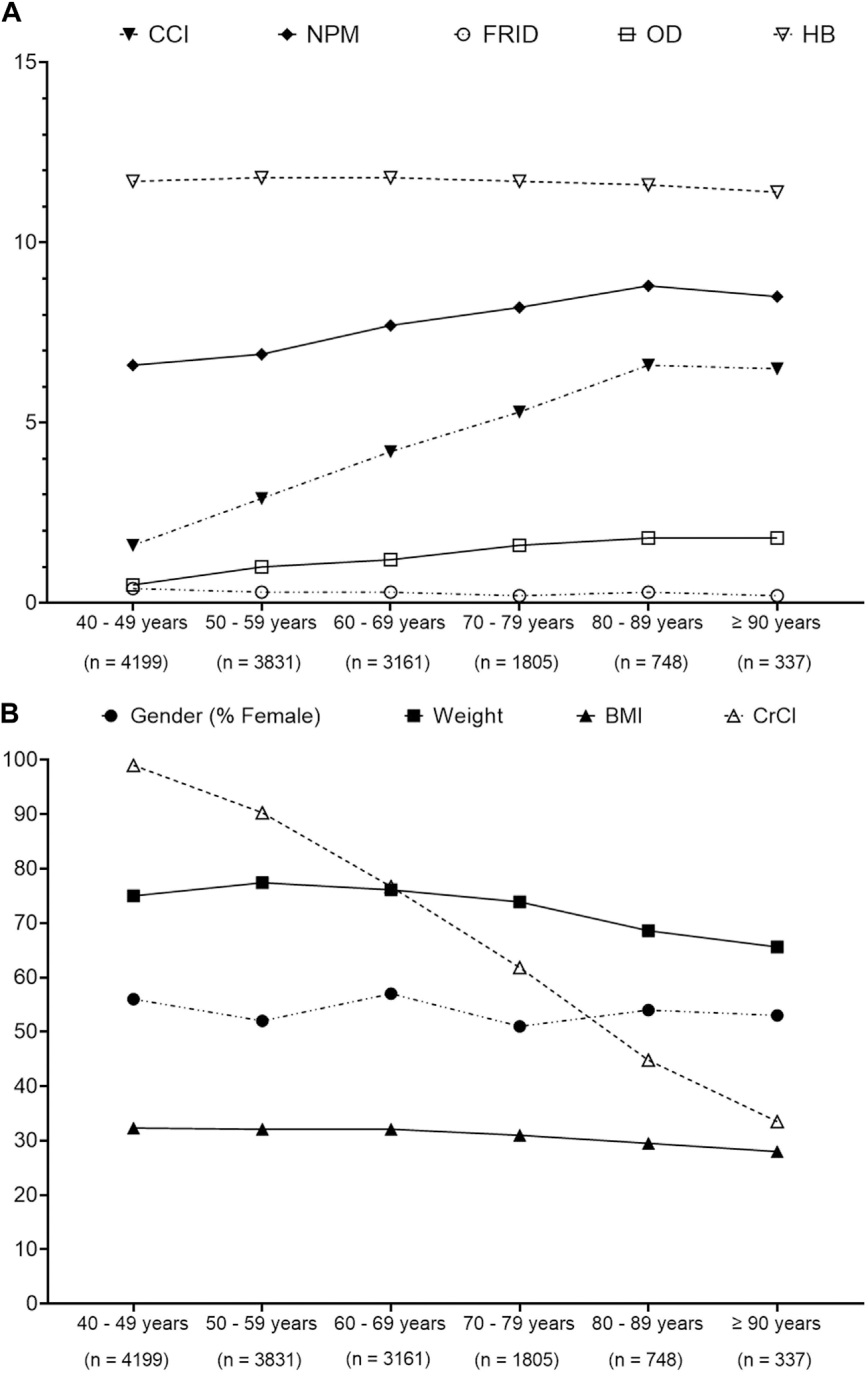
ANOVA analysis to determine the trends pattern of patients’ characteristics over different age groups. **(A)** Trends of Charlson comorbidity index (CCI), number of prescribed medication (NPM), fall risk increasing drugs (FRIDs), orthostatic hypotension-contributing drug (OD), and hemoglobin (HB). **(B)** Trends of gender, weight, body mass index (BMI), and creatinine clearance (CrCl). Data presented are the actual value.

Figure 2 presents that CCI, NPM, and OD medications expressed an increasing trend with increasing age among different age groups, whereas CrCl, weight, and FRID medication showed a decreasing trend.

Table 2 shows that female individuals were more likely to have polypharmacy (54% vs*.* 46%, *p* < 0.001) and hyper-polypharmacy 61% vs*.* 39%, *p* < 0.001) compared to male individuals. Hyper-polymedicated patients were older (61 vs*.* 58.8 vs. 56.4, *p* < 0.001), had a higher median CCI score (5 vs*.* 3 vs*.* 2, *p* < 0.001), a lower average CrCl value (77.8 vs*.* 83.5 vs*.* 88.3, *p* < 0.001), a lower average hemoglobin level (11.6 vs*.* 11.7 vs. 11.8, *p* < 0.001), and lower body weight (74.8 vs*.* 75 vs*.* 75.7, *p* < 0.001) compared to polymedicated or non-polymedicated patients, respectively. The average amount of FRID medications and the median amount of OD were higher among hyper-polymedicated patients compared to polymedicated and non-polymedicated patients ((0.7 vs*.* 0.2 vs*.* 0.1, *p* < 0.001) and (1 vs*.* 0 vs*.* 0, *p* < 0.001), respectively). Table 2 also demonstrates that the majority of the hyper-polymedicated patients were presented to an internal medicine care unit (38% vs*.* 31% vs*.* 25%, *p* < 0.001) compared to other care units, including an endocrinology (8% vs*.* 7% vs*.* 7%, *p* < 0.001), a hematology (7% vs*.* 4% vs*.* 3%, *p* < 00.1), and a pulmonary unit (8% vs*.* 3% vs*.* 1%, *p* < 0.001), compared to polymedicated or non-polymedicated patients. On the other hand, the majority of the polymedicated patients were presented to a rheumatology care unit (19% vs*.* 14% vs*.* 15%, *p* < 0.001) and a neurology care unit (10% vs*.* 7% vs*.* 9%, *p* < 0.001) compared to hyper-polymedicated or non-polymedicated patients.

**TABLE 2:** Characteristics of the cohort classified based on the polypharmacy level. BMI, body mass index; CCI, Charlson comorbidity index; NPM, number of prescribed medications; FRIDs, falls risk increasing drugs; ODs, orthostatic hypotension-contributing drugs; CrCl, creatinine clearance; HB, hemoglobin.

Characteristic	Cohort	Not polymedicated	Polymedicated	Hyper-polymedicated	*p*-value
	*n* = 14,081	*n* = 4,828 (34)	*n* = 4,953(35%)	*n* = 4,300 (%31)	
Gender (male), n (%)	6,430 (46%)	2,494 (52%)	2,261 (46)	1,675 (39)	
Gender (female), n (%)	7,651 (54%)	2,334 (48%)	2,692 (54%)	2,625 (61%)	<0.001
Age (years), mean (SD)	58.7 (13.2)	56.4 (12.5)	58.8 (13.2)	61 (13.6)	<0.001
Weight (Kg), mean (SD)	75.2 (18.1)	75.7 (18.4)	75 (17.6)	74.8 (18.2)	0.04
BMI (Kg/m^2^), mean (SD)	31.8 (15)	33 (16.8)	32.1 (15.2)	30.1 (12.2)	<0.001
CCI, median (IQR)	3 (1–7)	2 (0–4)	3 (1–6)	5 (2–8)	<0.001
NPM, median (IQR)	6 (2–15)	3 (1–4)	7 (5–9)	14 (10–15)	<0.001
FRID, median (IQR)	0 (0–1)	0 (0–0)	0 (0–1)	0 (0–2)	
FRID, mean (SD)	0.3 (0.7)	0.1 (0.2)	0.2 (0.5)	0.7 (0.9)	<0.001
OD, median (IQR)	1 (0–3)	0 (0–1)	0 (0–3)	1 (0–4)	<0.001
CrCl (mL/min), mean (SD)	83.4 (48.9)	88.3 (51.6)	83.5 (47.6)	77.8 (46.4)	<0.001
HB (g/dL), mean (SD)	11.7 (2.3)	11.8 (2.3)	11.7 (2.3)	11.6 (2.2)	0.002
Care unit specialty					
Internal medicine, n (%)	4,355 (31)	1,189 (25)	1,526 (31)	1,640 (38)	<0.001
Rheumatology, n (%)	2,301 (16)	737 (15)	962 (19)	602 (14)	<0.001
Cardiology, n (%)	1,282 (9)	458 (10)	477 (10)	347 (8)	<0.001
Neurology, n (%)	1,235 (9)	453 (9)	500 (10)	282 (7)	<0.001
Endocrinology, n (%)	1,037 (7)	358 (7)	348 (7)	331 (8)	<0.001
Urology, n (%)	781 (6)	633 (13)	116 (2)	32 (1)	<0.001
Gastroenterology, n (%)	697 (5)	373 (8)	212 (4)	112 (3)	<0.001
Hematology, n (%)	596 (4)	130 (3)	186 (4)	280 (7)	<0.001
Psychiatric, n (%)	618 (4)	281 (6)	255 (5)	82 (2)	<0.001
Pulmonary, n (%)	574 (4)	63 (1)	150 (3)	361 (8)	<0.001
Infectious disease, n (%)	385 (3)	80 (2)	158 (3)	147 (3)	<0.001
Nephrology, n (%)	219 (2)	72 (2)	63 (1)	84 (2)	<0.001

Data show that patients who attended pulmonary care units were prescribed the highest average NPM (average = 11 medications), followed by hematology (9 medications), infectious diseases (8.5 medications), internal medicine (8.1 medications), and nephrology (8 medications) care units (Supplementary Material).

The recorded comorbidities among the cohort are summarized in Table 3. The most recorded comorbidities in our study are osteoarthritis (OA; 37%), ischemic heart diseases (IHD; 34%), osteoporosis (OP; 33%), hypertension (HTN; 30%), and gastroesophageal reflux disease (GORD; 30%). The middle-aged patients had more OA (40% vs*.* 34%, *p* < 0.001), rheumatoid arthritis (RA) (24% vs*.* 15%, *p* < 0.001), and anemia (27% vs*.* 25%, *p* < 0.001) than older patients, and the older patients had more IHD (51% vs*.* 26%, *p* < 0.001), HTN (47% vs*.* 23%, *p* < 0.001), GORD (38% vs*.* 26%, *p* < 0.001), and hyperlipidemia (43% vs*.* 19%, *p* < 0.001) than middle-aged patients (Supplementary Material). Table 3 demonstrates that hyper-polymedicated patients had more GORD (60% vs*.* 25% vs*.* 10%, *p* < 0.001)), OA (59% vs*.* 36% vs*.* 20%, *p* < 0.001), OP (58% vs*.* 34% vs*.* 9%, *p* < 0.001)), and IHD (52% vs*.* 35% vs*.* 17%, *p* < 0.001) compared to polymedicated or non-polymedicated patients, respectively.

**TABLE 3:** Common recorded morbidities among the cohort classified based on the polypharmacy level. GORD, gastroesophageal reflux disease.

Comorbidity	Cohort	Not polymedicated	Polymedicated	Hyper-polymedicated	*p*-value
	*n* = 14,081	*n* = 4,828	*n* = 4,953	*n* = 4,300	
Osteoarthritis, n (%)	5,262 (37)	987 (20)	1,757 (36)	2,518 (59)	<0.001
Ischemic heart disease, n (%)	4,783 (34)	812 (17)	1,718 (35)	2,253 (52)	<0.001
Osteoporosis, n (%)	4,616 (33)	435 (9)	1,699 (34)	2,482 (58)	<0.001
Hypertension, n (%)	4,279 (30)	632 (13)	1,571 (32)	2,076 (48)	<0.001
GORD, n (%)	4,209 (30)	476 (10)	1,212 (25)	2,521 (60)	<0.001
Hyperlipidemia, n (%)	3,738 (27)	395 (8)	1,454 (29)	1,889 (44)	<0.001
Anemia, n (%)	3,726 (27)	481 (10)	1,157 (23)	2,088 (49)	<0.001
Heart failure, n (%)	3,549 (25)	499 (10)	1,324 (27)	1,726 (40)	<0.001
Diabetes mellitus, n (%)	2,993 (21)	321 (7)	1,044 (21)	1,628 (38)	<0.001
Rheumatoid arthritis, n (%)	2,970 (21)	485 (10)	1,046 (21)	1,439 (34)	<0.001

**FIGURE 3:**
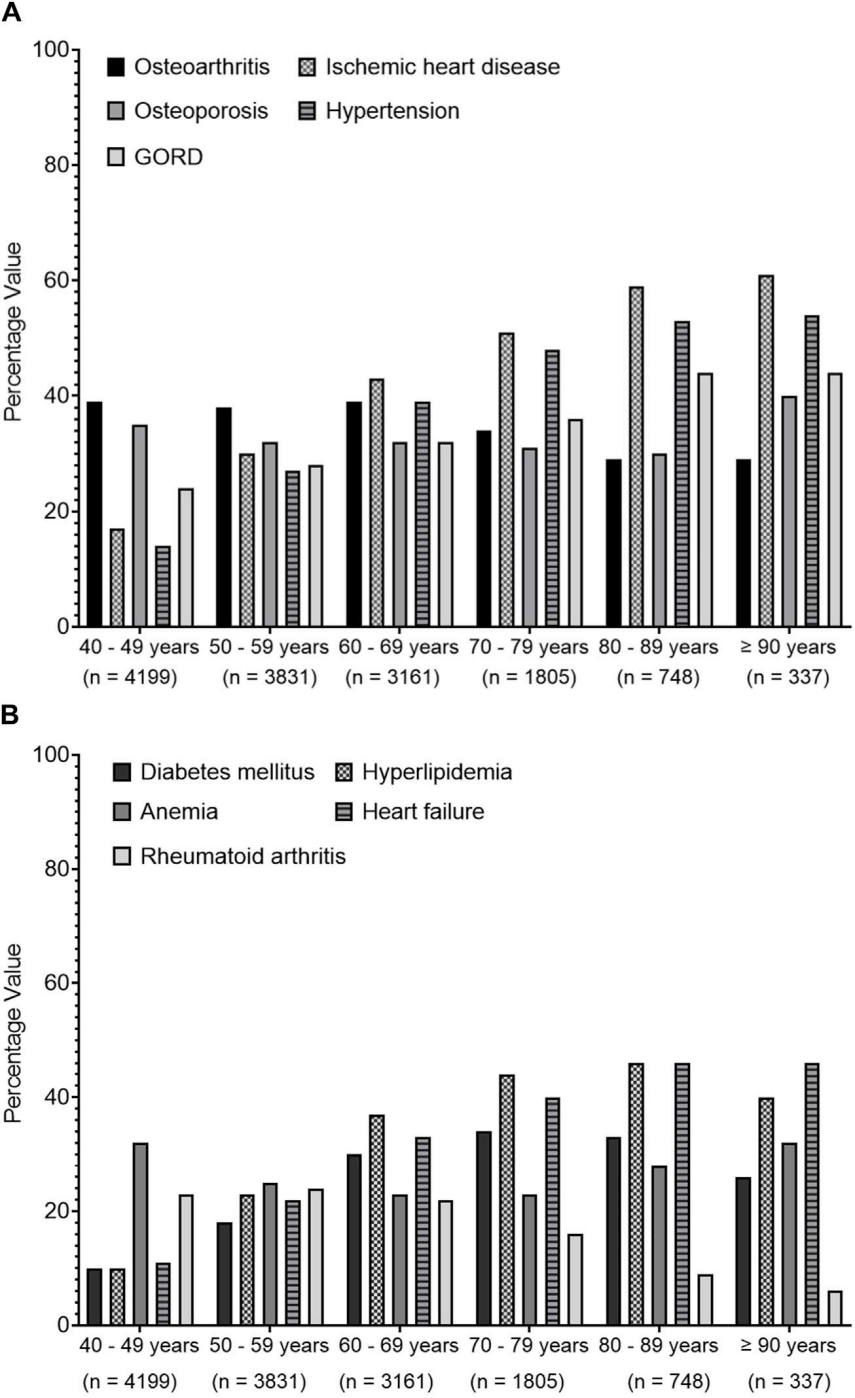
Trends of common recorded comorbidities over different age groups. Data present the percentage value of the referenced group. **(A)** presents data for osteoarthritis, ischemic heart diseases, osteoporosis, hypertension and gastroesophageal reflux disease (GORD), **(B)** represents data for diabetes mellitus, hyperlipidaemia, anemia, heart failure and rheumatoid arthritis.

Figure 3 shows that there was an increasing prevalence trend of IHD, HTN, hyperlipidemia, and heart failure (HF) with increasing age. OA and OP shared a similar trend pattern, where their trends were fluctuating between the age groups from 40 to 69 years before expressing a decreasing trend. However, RA presented a decreasing trend with increasing age. Diabetes mellitus (DM) showed an increasing trend until the age group 70–79 years, which then expressed a decreasing trend.

In the internal medicine care unit, the most common comorbidities recorded were HF (50%), HTN (45%), DM (45), and IHD (43). As expected, RA (49%) was the most common comorbidity recorded in the rheumatology care unit (Supplementary Material).

The most commonly prescribed medication classes to the cohort were medications related to the alimentary system and metabolism (57%), musculoskeletal system (48%), nervous system (46%), blood and blood-forming organs (42%), and cardiovascular system (42%) (Supplementary Material). Older patients were prescribed more medications related to the alimentary system and metabolism (66% vs*.* 53%, *p* < 0.001) and blood and blood-forming organs (52% vs*.* 38%, *p* < 0.001) but less medications related to the musculoskeletal system (50% vs*.* 43%, *p* < 0.001) and nervous system (48% vs*.* 43%, *p* < 0.001) compared to middle-aged patients (Supplementary Material). Hyper-polymedicated patients have more prescribed medications related to the alimentary system and metabolism (89% vs*.* 61% vs*.* 25%, *p* < 0.001), musculoskeletal system (72% vs*.* 50% vs*.* 25%, *p* < 0.001), nervous system (68% vs*.* 49% vs*.* 25%, *p* < 0.001), blood and blood-forming organs (70% vs*.* 43% vs*.* 17%, *p* < 0.001), and cardiovascular system (62% vs*.* 43% vs*.* 23%, *p* < 0.001) than polymedicated and non-polymedicated patients, respectively (Table 4).

**TABLE 4:** Prevalence of medications prescribed among the cohort classified based on the polypharmacy level. Medications were presented by the first-level order as per the anatomical therapeutic classification.

Medication class	Cohort	Not polymedicated	Polymedicated	Hyper-polymedicated	*p*-value
	*n* = 14,081	*n* = 4,828	*n* = 4,953	*n* = 4,300	
Alimentary and metabolism, n (%)	8,043 (57)	1,211 (25)	2,023 (61)	3,809 (89)	<0.001
Musculoskeletal system, n (%)	6,782 (48)	1,189 (25)	2,494 (50)	3,099 (72)	<0.001
Nervous system, n (%)	6,528 (46)	1,197 (25)	2,403 (49)	2,928 (68)	<0.001
Blood and blood-forming organs, n (%)	5,943 (42)	796 (17)	2,138 (43)	3,009 (70)	<0.001
Cardiovascular system, n (%)	5,880 (42)	1,097 (23)	2,130 (43)	2,653 (62)	<0.001
Systemic hormonal preparation, n (%)	2,081 (15)	323 (7)	589 (12)	1,169 (27)	<0.001
Antineoplastic and immuno-modulating agents, n (%)	1,506 (11)	85 (2)	450 (9)	971 (23)	<0.001
Genito-urinary system and sex hormones, n (%)	1,417 (10)	651 (14)	332 (7)	434 (10)	<0.001
Respiratory system, n (%)	1,457 (10)	51(1)	228 (5)	1,178 (27)	<0.001
Anti-infective for systemic use, n (%)	1,372 (10)	208 (4)	338 (7)	826 (19)	<0.001
Sensory organs, n (%)	643 (5)	70 (1)	149 (3)	424 (10)	<0.001
Dermatological, n (%)	427 (3)	44 (1)	52 (1)	331 (8)	<0.001
Various, n (%)	219 (2)	4 (0.1)	29 (0.6)	186 (4.3)	<0.001
Antiparasitic products, insecticides, and repellents, n (%)	127 (1)	2 (0.1)	17 (0.3)	108 (3)	<0.001

**FIGURE 4:**
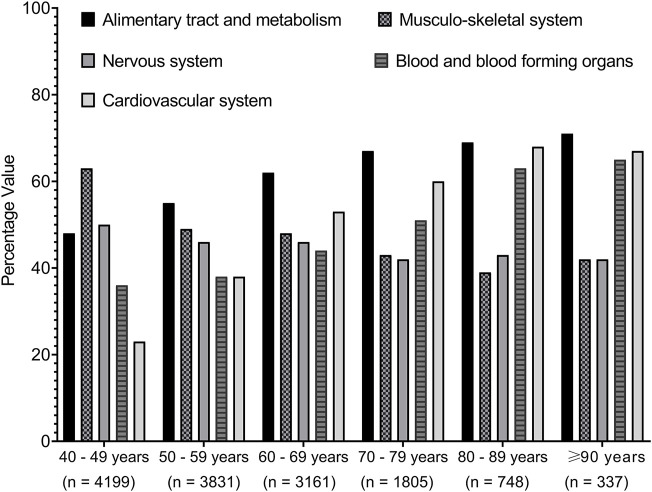
Trends of medications prescribed among the cohort classified based on the age groups. Medications were presented by the first-level order as per the anatomical therapeutic classification. Data present the percentage value of the referenced group.

Next, we investigated the impact of age on the different medication classes. Figure 4 shows that there was an increasing prevalence trend of prescribing medications related to the cardiovascular system, blood and blood-forming organs, and alimentary system and metabolism, but a decreasing trend was seen for those related to the musculoskeletal system and nervous system with increasing age over different age groups.

Furthermore, we decided to determine the most common prescribed medication in patients with polypharmacy. We found that proton pump inhibitors (PPIs) (30%), non-steroidal anti-inflammatory drugs (NSAIDs) (30%), vitamin D (30%), and statins (26%) were the most prescribed medications among the cohort (Supplementary Material). Older patients were prescribed more PPIs (38% vs*.* 26%, *p* < 0.001), statins (43% vs*.* 19%, *p* < 0.001), and antiplatelet aggregation drugs (37% vs*.* 19%, *p* < 0.001) and fewer NSAIDs (24% vs*.* 31% *p* < 0.001) and vitamin D (31% vs*.* 27%, *p* < 0.001) compared to middle-aged patients. Similarly, hyper-polymedicated patients were prescribed more PPIs (59% vs*.* 25% vs*.* 10%, *p* < 0.001), NSAIDs (45% vs*.* 27% vs*.* 16%, *p* < 0.001), vitamin D (52% vs*.* 32% vs*.* 8%, *p* < 0.001), and statins (44% vs*.* 29% vs*.* 8%, *p* < 0.001) compared to polymedicated and non-polymedicated patients, respectively (Table 5).

**TABLE 5:** Prevalence of medications prescribed among cohort classified based on the polypharmacy level. Medications were presented based on their therapeutics class. NSAIDs, non-steroidal anti-inflammatory drugs; ACEIs, angiotensin-converting enzyme inhibitors.

Medication class	Cohort	Not polymedicated	Polymedicated	Hyper-polymedicated	*p*-value
	*n* = 14,081	*n* = 4,828	*n* = 4,953	*n* = 4,300	
Proton pump inhibitors, n (%)	4,209 (30)	476 (10)	1,212 (25)	2,521 (59)	<0.001
NSAIDs, n (%)	4,059 (30)	773 (16)	1,345 (27)	1,941 (45)	<0.001
Vitamin D, n (%)	4,178 (30)	390 (8)	1,574 (32)	2,214 (52)	<0.001
Statin, n (%)	3,711 (26)	385 (8)	1,445 (29)	1,881 (44)	<0.001
Antiplatelet, n (%)	3,018 (21)	291 (6)	1,167 (24)	1,560 (36)	<0.001
Calcium channel blockers, n (%)	2,358 (17)	296 (6)	807 (16)	1,255 (29)	<0.001
Beta-blockers, n (%)	2,417 (17)	365 (8)	857 (17)	1,195 (28)	<0.001
Anticoagulants, n (%)	1,770 (13)	202 (4)	462 (9)	1,106 (26)	<0.001
ACEIs, n (%)	1,783 (13)	241 (5)	731 (15)	811 (19)	<0.001
Diuretics, n (%)	1,737 (12)	141 (3)	564 (11)	1,032 (24)	<0.001

**FIGURE 5:**
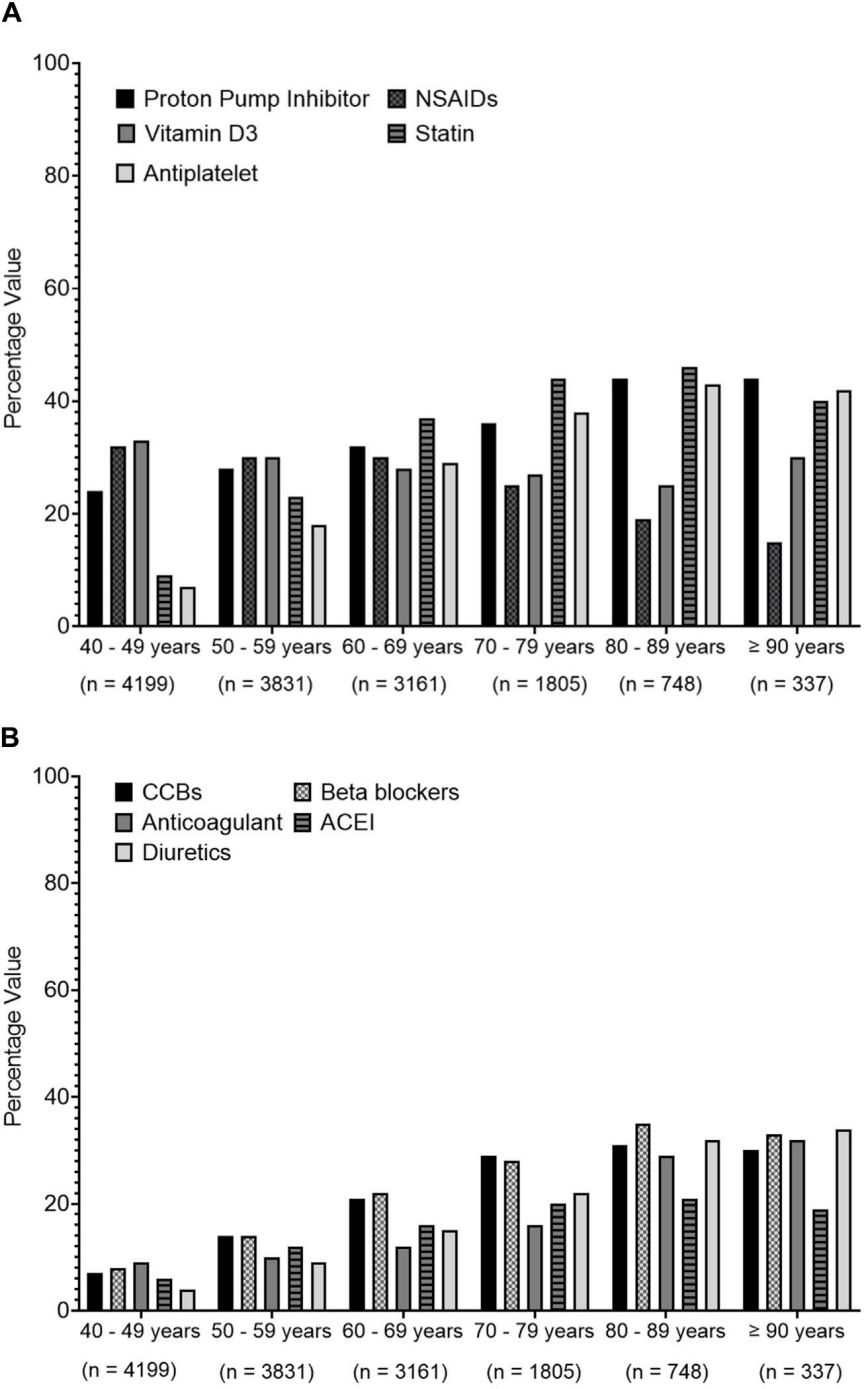
Prescribing trends of commonly prescribed medications over different age groups. Medications were presented based on their therapeutics class. **(A)** presents data for proton pump inhibitors, non-steroidal anti-inflammatory drugs (NSAIDs), vitamin D3, statins, anti-platelets, **(B)** represents data for calcium channel blockers (CCBs), beta blockers, anti-coagulants, angiotensin converting enzyme inhibitors (ACEIs), diuretic.

Regarding the association between age and the prescribed medications in polypharmacy patients, Figure 5 shows an increasing prescription trend for PPIs, statins, and other cardiovascular system medications such as calcium channel blockers (CCBs), beta-blockers (BBs), angiotensin-converting enzyme inhibitors (ACEIs), diuretics, antiplatelets, and anticoagulants, but a decreasing prescribing trend for NSAIDs with increasing age is seen among different age groups.

Finally, we determined the most common prescribed medications in the different specialty care units. The analysis shows that most of the patients who attended a pulmonary (58%), gastroenterology (46%), and internal medicine (38%) care unit were prescribed PPIs. Additionally, 70% of the patients who attended a rheumatology care unit and 42% of those who attended a hematology care unit were prescribed NSAIDs. We also found that diuretics were prescribed more for patients who attended the nephrology care unit (34%) and the cardiology care unit (33%) (Supplementary Material).

Interestingly, polypharmacy was negatively associated with increasing age among the cohort, older patients, and middle-aged patients ((OR = 0.9, 95% CI 0.89–0.91), (OR = 0.9, 95% CI 0.89–0.92), and (OR = 0.9, 95% CI 0.85–0.95), respectively), but highly associated with prescribing FRID ((OR = 9.7, 95% CI 8.5–11.2), (OR = 11.2, 95% CI 8.2–15.3), and (OR = 9.5, 95% CI 8.1–11.1), respectively) and with CCI ((OR = 3.4, 95% CI 3.3–3.6), (OR = 3.7, 95% CI 3.4–4.1), and (OR = 3.4, 95% CI 3.2–3.6), respectively) (Table 6). The highest medication class associated with polypharmacy among the cohort, older patients, and middle-aged patients included nervous system medications ((OR = 4.5, 95% CI 4–5), (OR = 4.8, 95% CI 3.8–6), and (OR = 4.5, 95% CI 4–5.1), respectively) and musculoskeletal system medications ((OR = 4.2, 95% CI 3.8–4.7), (OR = 6, 95% CI 4.8–7.6), and (OR = 3.8, 95% CI 3.3–4.2), respectively). Osteoporosis was the highest associated morbidity with polypharmacy among the cohort, older patients, and middle-aged patients ((OR = 6, 95% CI 5.4–7), (OR = 9.3, 95% CI 6.9–12.5), and (OR = 5.5, 95% CI 4.7–6.3), respectively).

**TABLE 6:** Multivariate analysis for factors associated with polypharmacy among the cohort, older patients, and middle-aged patients. Data were generated from binary logistic regression and presented as odds ratio (OR) and 95% confidence interval (95% CI); the model was adjusted for age, gender, Charlson comorbidity index, falls risk increasing drugs, orthostatic hypotension-contributing drugs, and creatinine clearance. GORD = gastroesophageal reflux disease.

Factor	Total cohort	Middle-aged adults	Older adults
	*n* = 14,081	*n* = 9,722	*n* = 4,359
Age	0.9 (0.89–0.91)	0.9 (0.85–0.95)	0.9 (0.89–0.92)
Gender (female)	1.3 (1.2–1.5)	1.4 (1.3–1.6)	1.2 (1.1–1.5)
Charlson comorbidity index	3.4 (3.3–3.6)	3.4 (3.2–3.6)	3.7 (3.4–4.1)
Falls risk increasing drugs	9.7 (8.5–11.2)	9.5 (8.1–11.1)	11.2 (8.2–15.3)
Orthostatic hypotension-contributing drugs	1.1 (1.05–1.2)	1.1 (0.95–1.2)	1.2 (1.1–1.3)
Creatinine clearance	0.99 (0.98–1.05)	1 (0.95–1.05)	0.95 (0.9–1.05)
Medication classes			
Alimentary tract and metabolism	3.8 (3.4–4.2)	3.4 (3–3.8)	5.3 (4.2–6.6)
Blood and blood-forming organs	3 (2.7–3.3)	2.9 (2.6–3.3)	2.9 (2.3–3.6)
Cardiovascular system	0.6 (0.5–0.7)	0.6 (0.5–0.7)	0.8 (0.6–1.1)
Musculoskeletal system	4.2 (3.8–4.7)	3.8 (3.3–4.2)	6 (4.8–7.6)
Nervous system	4.5 (4–5)	4.5 (4–5.1)	4.8 (3.8–6)
Comorbidities			
GORD	1.4 (1.3–1.6)	1.3 (1.2–1.6)	1.6 (1.2–2.1)
Diabetes mellitus	1.3 (1.1–1.5)	1.3 (1.1–1.5	1.4 (1.1–1.8)
Hypertension	0.25 (0.2–0.3)	0.2 (0.15–0.3)	0.3 (0.2–0.4)
Ischemic heart disease	0.1 (0.05–0.15)	0.1 (0.05–0.15)	0.1 (0.05–0.2)
Hyperlipidemia	2.5 (2.2–2.9)	2.2 (1.9–2.6)	3.3 (2.6–4.2)
Osteoarthritis	2.5 (2.2–2.7)	2.3 (2–2.6)	3.4 (2.7–4.3)
Osteoporosis	6 (5.4–7)	5.5 (4.7–6.3)	9.3 (6.9–12.5)
Rheumatoid arthritis	2.7 (2.4–3.1)	2.5 (2.2–2.9)	4.1 (3–5.6)

## 4 Discussion

The results of this study provided insight into the extent of polypharmacy, hyper-polypharmacy, and the prescribing patterns in middle-aged and older patients in the Eastern Region, Saudi Arabia. The prevalence of polypharmacy was 66% among the cohort, whereas 31% of the cohort was exposed to hyper-polypharmacy. Patients presenting with no polypharmacy were heavier and were prescribed a lower amount of FRID medications compared to polymedicated or hyper-polymedicated patients. Female patients were more prescribed medications and were more exposed to polypharmacy than male patients. The majority of the polymedicated patients presented to internal medicine or rheumatology care units and suffered more from diseases related to musculoskeletal disorders or cardiovascular disorders. NSAIDs and PPIs were the most prescribed drugs within the cohort. The prevalence of polypharmacy was negatively associated with aging and the concurrent prescribing of cardiovascular system medications and positively associated with increasing CCI and the concurrent prescribing of musculoskeletal system or alimentary tract and metabolism system medications.

The major finding of the current study is that 66% of the cohort was exposed to polypharmacy or hyper-polypharmacy (35% and 31%, respectively). This value is considered high compared to a previous study from Saudi Arabia, which reported that the prevalence of polypharmacy was 46.5% (Balkhi et al., 2021). This prevalence is also high compared to a recently published study from Australia, in which the prevalence of polypharmacy was 36.1% (Page et al., 2019). One reason for the increased prevalence in our study could be related to the nature of the study cohort as this study included patients who attended a medical care unit. Balkhi et al. included patients aged 18 years and above who attended any outpatient clinic from a different care setting (Balkhi et al., 2021), whereas Page et al. collected dispensing data for patients aged 70 years and above from the Pharmaceutical Benefits Scheme (PBS) system (Page et al., 2019). It is important to indicate that as chronic diseases are usually first reported in the middle age, particularly after exposure to unhealthy lifestyles and as a result of chronic complex inflammation (Prasad et al., 2012), findings generated from this study set it as an advantage over other studies. Most of the patients (young or old) with chronic morbidities would attend a medical care unit to manage their comorbidities, and the current research clearly describes the prevalence of polypharmacy among patients who attended such care units (Prasad et al., 2012).

The second finding from this study is that the prevalence of polypharmacy among older patients was higher compared to that in middle-aged patients (73% vs. 62%, *p* < 0.001). This finding was consistent with previous studies reported from Saudi Arabia (Alwhaibi et al., 2018a; Alwhaibi et al., 2020; Balkhi et al., 2021). Although hyper-polypharmacy was more common among older patients compared to middle-aged patients (38% vs. 27%, *p* < 0.001), it is important to pay close attention to the reported value of polypharmacy and hyper-polypharmacy among the middle-aged patients. Another advantage of the current study is that no previous study has reported a similar finding in Saudi Arabia. This finding alerts toward the importance of assessing the risk of polypharmacy and its consequences when planning pharmaceutical care for middle-aged patients as it remains high and should be reviewed.

Fascinatingly, this study showed a negative association between polypharmacy and aging, which is inconsistent with previous reports where polypharmacy was six times higher in patients aged ≥61 years compared to those ≤60 years (Salih et al., 2013), or it was seven times higher in older diabetic patients compared to middle-aged or younger patients (Alwhaibi et al., 2018a). This finding shows aging is not as salient a factor contributing to polypharmacy as other factors, such as gender or CCI. An explanation could be the good practice of the prescribing physician in the hospital where the study was conducted to reduce polypharmacy and its consequences among older patients by applying a de-prescribing approach (Thompson et al., 2019).

Findings generated from the current study emphasize the importance of reviewing the appropriateness of FRID medications, particularly because they were prescribed for middle-aged patients more often than older patients. In addition, hyper-polymedicated patients had lower body weights and were more likely to consume FRID medications compared to polymedicated or non-polymedicated patients (Table 2). Trevisan et al. suggested that the use of FRID medications may lead to falls, which results in weight decline; hence, FRID medication is an indirect cause of low body weight (Trevisan et al., 2021).

The third finding of this research is that female patients were more often exposed to polypharmacy compared to male patients. The finding is consistent with previous studies generated from Saudi Arabia (Salih et al., 2013; Alwhaibi et al., 2018a) and internationally (Nobili et al., 2011; Page et al., 2019). The possible interpretation is that female individuals generally report more diseases and seek healthcare for their health conditions more often compared to male individuals (Al-Qurain et al., 2020b). This finding is crucial when caring for a polymedicated female patient as polypharmacy reflects a high risk of inappropriate use of medications and ADR (Sapkota et al., 2011; Assari et al., 2019). Joung et al. reported that diabetic female patients reported ADRs related to their antidiabetic medications more often compared to diabetic male patients (Joung et al., 2020).

The fourth finding is that there were positive associations between the prevalence of polypharmacy and increasing CCI. This is consistent with the previous studies, where drug prescriptions increased along with the increasing number of morbidities (Alsuwaidan et al., 2019a; Menditto et al., 2019). Our finding confirms that the presence of multiple comorbidities is a key contribution to polypharmacy and not aging. It could enhance the awareness of the practice toward the importance of setting policies to detect polypharmacy at an early age in order to avoid its later consequences in the patient’s life. Previous reports linked polypharmacy with frailty, but due to the nature of data collection, CCI was used as a predictor of a patient’s condition and a substitute for frailty in this study (Al-Qurain et al., 2020a).

Previous studies have stated that heart diseases and HTN were risk factors for polypharmacy (Jyrkkä et al., 2009; Khezrian et al., 2020). Surprisingly, the current study found a negative association between the prevalence of polypharmacy and having HTN or IHD (OR = 0.25 and 0.1, respectively). In contrast, polypharmacy was positively associated with having OP, OA, hyperlipidemia, or GORD (OR = 6, 2.5, 2.5, and 1.4, respectively). The main reason for these differences could be related to the nature of the cohort included, as described previously. Our results indicated that the prevalence of OA was higher among middle-aged patients compared to the older patients (40% vs. 34%, *p* < 0.001), which is inconsistent with a previous study where polypharmacy was common in OA patients, and it was associated with a worse health status in previous reports (Betancourt et al., 2022). This triggers concerns regarding the risk of worsening health status associated with polypharmacy. This could be a reason why older patients had a higher prevalence of GORD compared to middle-aged as this could be linked to the risk of ADRs associated with using medications to manage OA in early life, such as NSAIDs.

Finally, several studies showed that polypharmacy was associated with prescribing medications related to cardiovascular diseases (Jyrkkä et al., 2009; Salih et al., 2013; Alwhaibi et al., 2018a). This is inconsistent with the current study as polypharmacy was negatively associated with prescribing cardiovascular system medication (OR = 0.6), but it was positively associated with prescribing alimentary tract and metabolism or musculoskeletal system medication (OR = 3.8 and 4.2, respectively). Particularly, NSAIDs (30%) and PPIs (30%) were the most prescribed medications reported in the current study. NSAIDs are commonly used to treat pain and inflammation related to OA, but they can cause several ADRs, namely, nephrotoxicity, cardiovascular events, and GI ulceration (Young et al., 2021). NSAIDs are also a common cause of DDIs. Medications including antiplatelets, corticosteroids, and anticoagulants can all increase the risk of GI bleeding if they are taken concurrently with NSAIDs, especially when they are taken with multiple other medications or used chronically (Wongrakpanich et al., 2018). Similarly, PPIs are commonly co-prescribed with NSAIDs to reduce the risk of GI bleeding. However, there are concerns regarding their long-term safety as they can cause fracture, pneumonia, and reduced iron, magnesium, and B12 absorption (Reimer, 2013).

There are several limitations in this study that should be acknowledged. First, it was not feasible to know the duration of medication use as data were collected retrospectively from patients’ medical records. This limited the ability to describe the appropriateness of medications and the interpretation of how effective these medications were. Second, follow-up data were not available, and thus, longer-term treatment outcomes, including efficacy, ADRs, and readmission rates, were not obtained. Third, as this was a retrospective study, comorbidities were obtained from reviewing medical records, but the Rx-Risk comorbidity index was applied to mitigate against omissions in medical records. Fourth, as the given data collection was retrospective, it was not always possible to determine the indication for all the prescribed medications. Determination and inclusion of as-needed medications may cause bias, which results in the overestimation of medication usage. Finally, this study was cross-sectional in nature, and thus, it was not possible to describe the trajectory of the mediations used over time and how these related to changes in the trajectories of disease progression, comorbidities, and frailty.

It is important to acknowledge that the COVID-19 pandemic could have had an impact on the data collected and the prevalence of polypharmacy. During the peak of COVID-19, patients may have been hesitant to visit healthcare facilities for non-urgent matters, potentially leading to changes in patient attendance and healthcare-seeking behavior. This could have influenced the population of patients included in the study and, subsequently, the prevalence of polypharmacy observed.

It is important to mention that the results presented in this study should not be interpreted to convey that some medications were used inappropriately. There were many valid indications for prescribing these medications, such as NSAIDs and PPIs. However, several comorbidities were presented that contraindicate the use of specific analgesic classes, such as pulmonary diseases, GORD, IHD, and renal impairment. The prevalence of hyper-polypharmacy, specifically in female older patients, should be evaluated as the risk of ADRs is expected to be higher, which could have an impact on the quality of life. Moreover, the prescription of NSAIDs and PPIs should be evaluated in middle-aged patients to reduce the risk of the prescribing cascade, which, in return, will reduce polypharmacy. Additionally, as polypharmacy is associated with a high risk of inappropriate prescriptions and an increased incidence of ADR, the role of pharmacists in medication review should be promoted (Sapkota et al., 2011; Assari et al., 2019; Baruth et al., 2020). Results generated from this study showed that polypharmacy can occur due to many reasons, and it is also imperative to note that due to the multiple comorbidities, there are also instances where polypharmacy is unavoidable (Dagli and Sharma, 2014). However, it has been noted that patients struggle with adherence in these situations (Kini and Ho, 2018). As a result, the utilization of medication organizer tools with the collaborative effort of the patient’s healthcare team, adherence, and safety can be assessed and potentially improved in those with a high medication burden.

Future research should investigate the duration of medication use and frailty level as part of a comprehensive approach to assess polypharmacy and its impact on patients’ health outcomes. There is a high need for further data to determine longer-term adverse effect outcomes and readmission rates among polymedicated patients.

## 5 Conclusion

Our findings suggest that polypharmacy is still an ongoing concern, not only in geriatric practice but also in general medical practice for middle-aged and older patients. Female patients were prescribed more medications and were exposed to polypharmacy and hyper-polypharmacy more often than male patients. NSAIDs and PPIs were the most prescribed drugs within the cohort. Polypharmacy was associated with increasing CCI and prescribing medications related to the musculoskeletal system or alimentary tract and metabolism system. With the collaborative effort of the patient’s healthcare team and the tools outlined above, patients’ commitment to the medication plan and safety can be evaluated, which can potentially improve the quality of life for the patients overburdened with higher medications.

## Data Availability

The raw data supporting the conclusion of this article will be made available by the authors, without undue reservation.
