# Supplementary material for: The prevalence of polypharmacy and hyper-polypharmacy among middle-aged vs. older patients in Saudi Arabia: a cross-sectional study
Source: Front Pharmacol. 2024 Jun 12;15:1357171. doi: 10.3389/fphar.2024.1357171 (PMC11200110; doi:10.3389/fphar.2024.1357171)
Supplement: Supplementary file 1 [file DataSheet1.docx]

**Supplementary Materials**

| 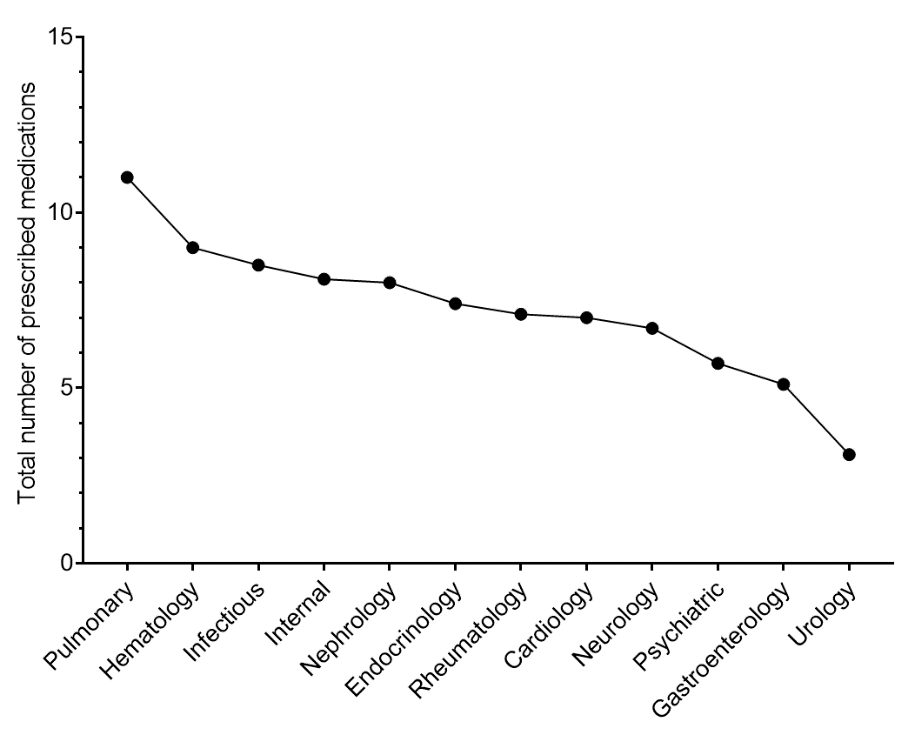 |
| --- |

**Figure SM_1: Total number of prescribed medications over each specialty care unit.**

| 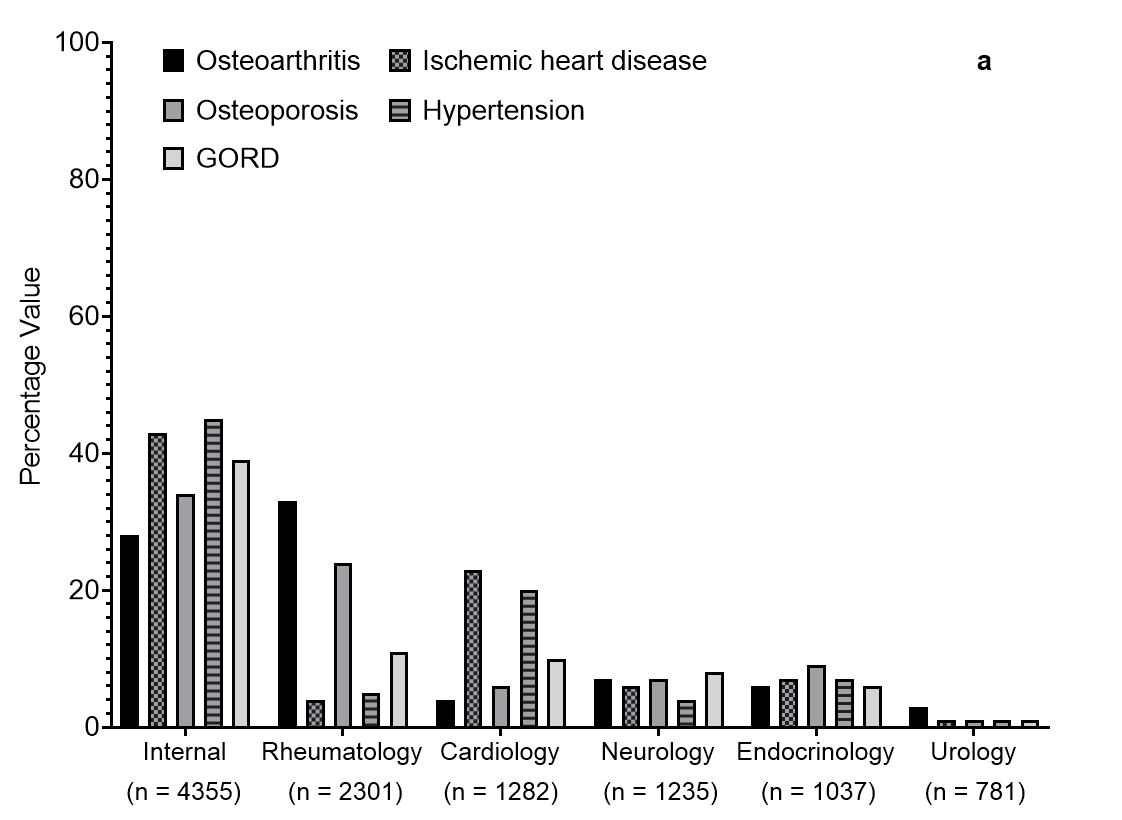 | 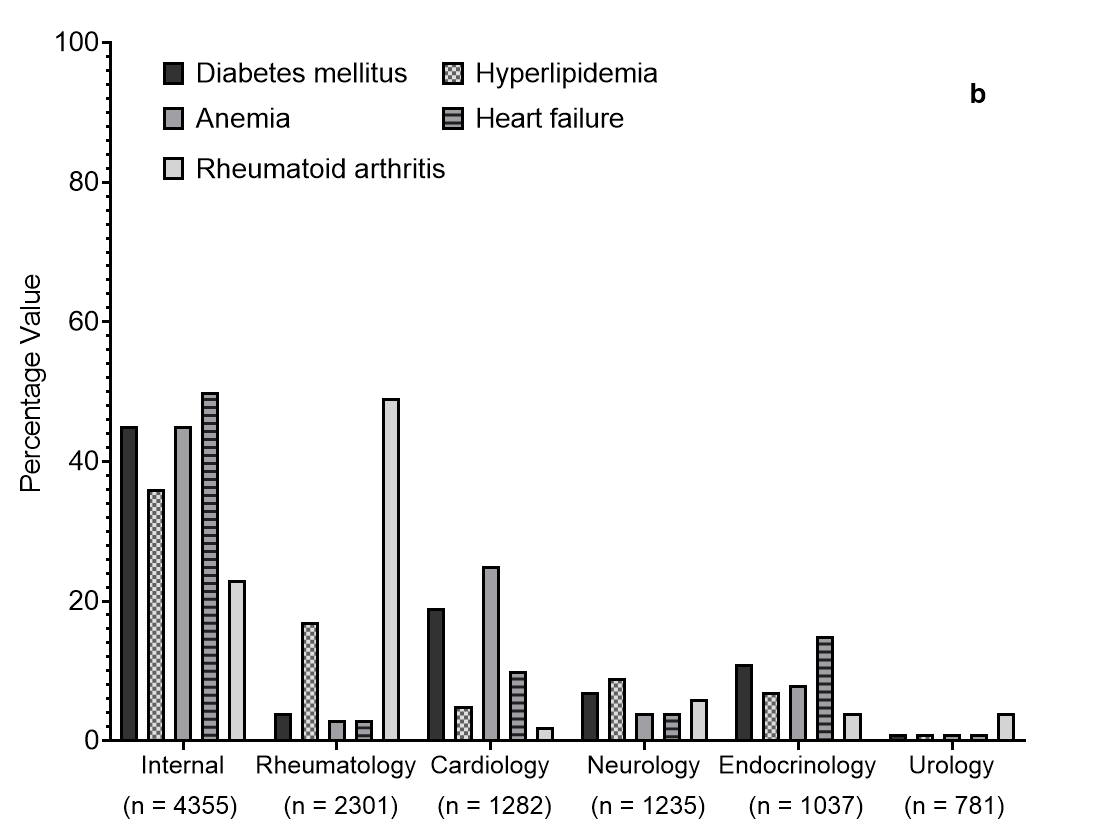 |
| --- | --- |
| 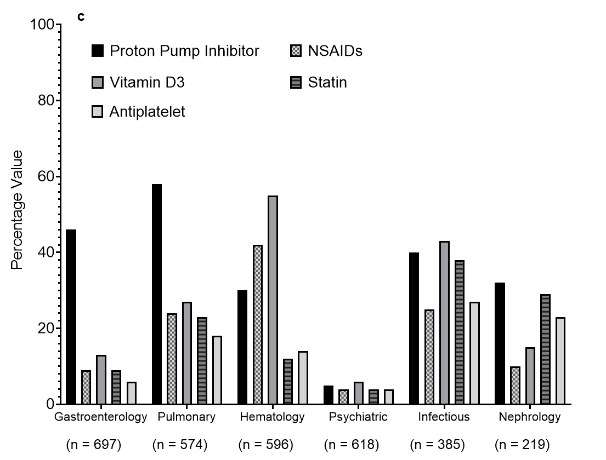 | 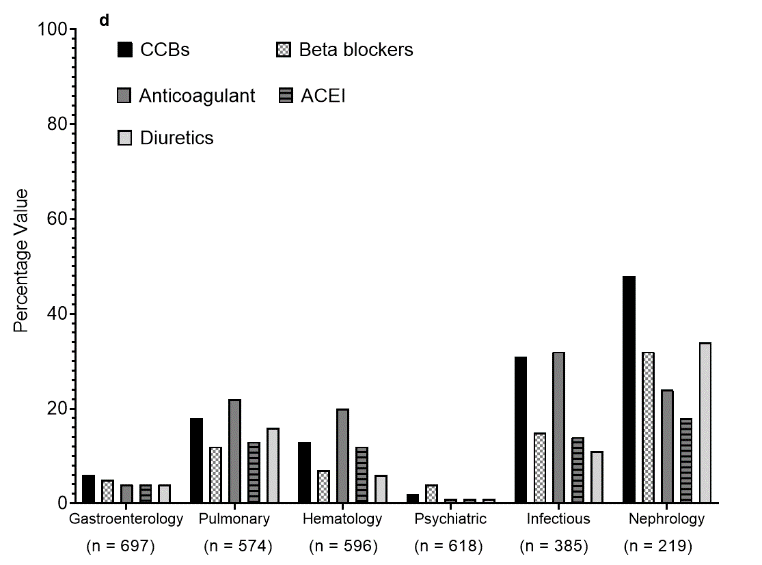 |

**Figure SM_2: (a) and (b) show the prevalence of common recorded comorbidities presented in the different specialty care units. (c) and (d) show the most commonly prescribed medication classes in the different specialty care units. Data presents the percentage value of the referenced group.**

| 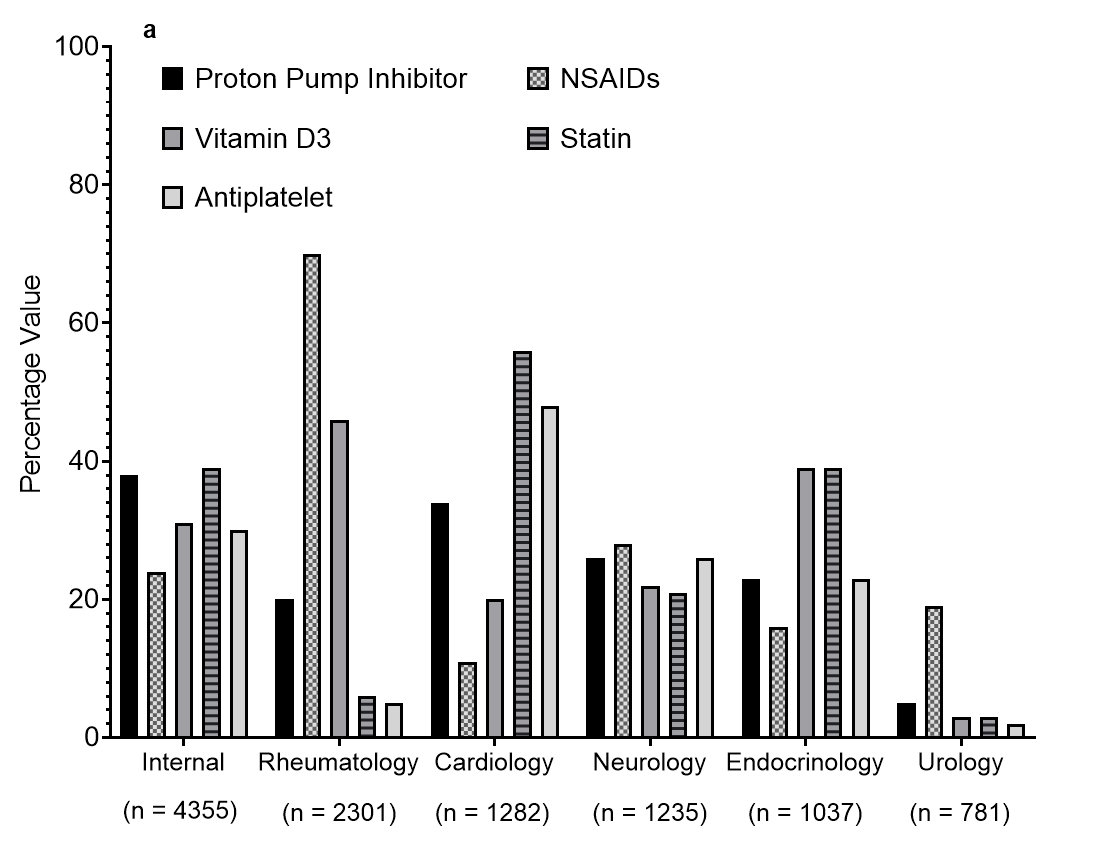 | 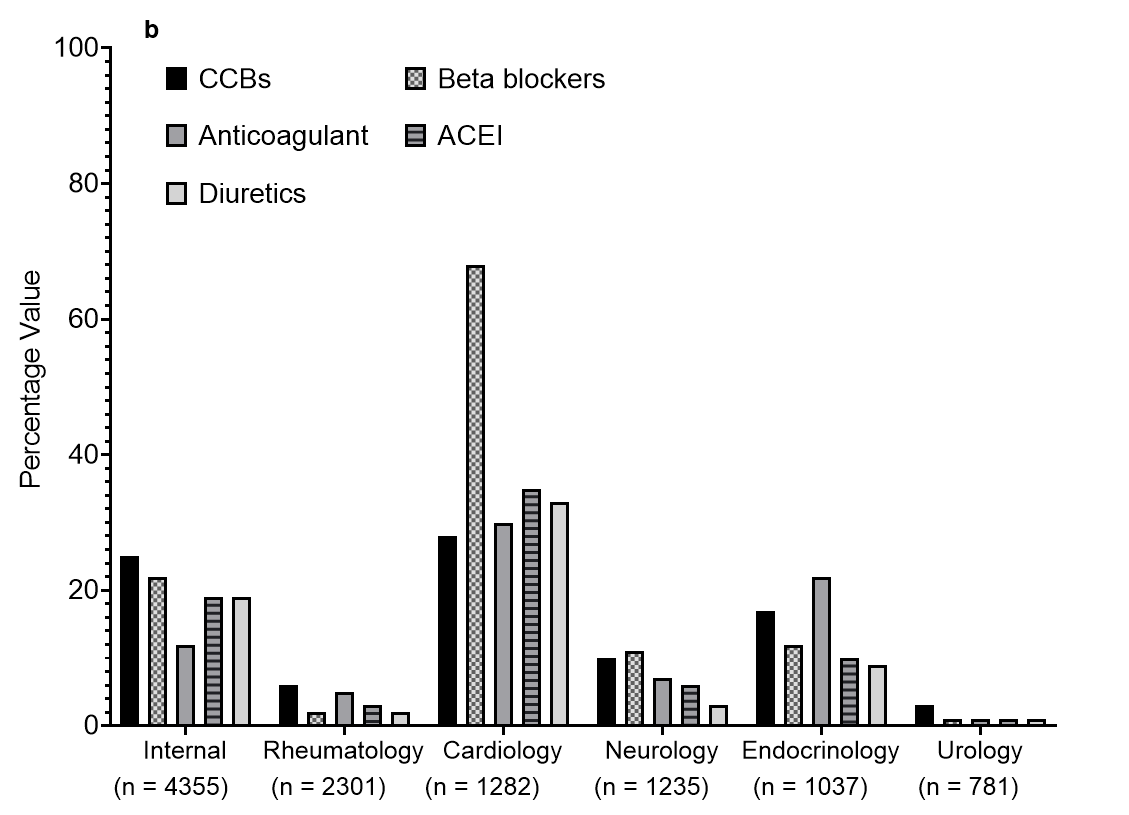 |
| --- | --- |
| 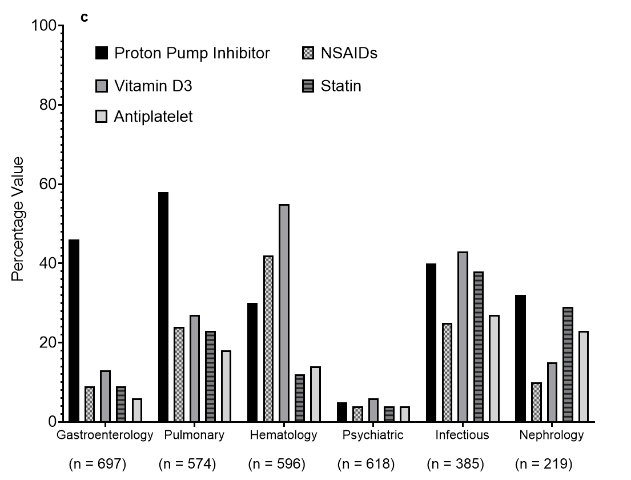 | 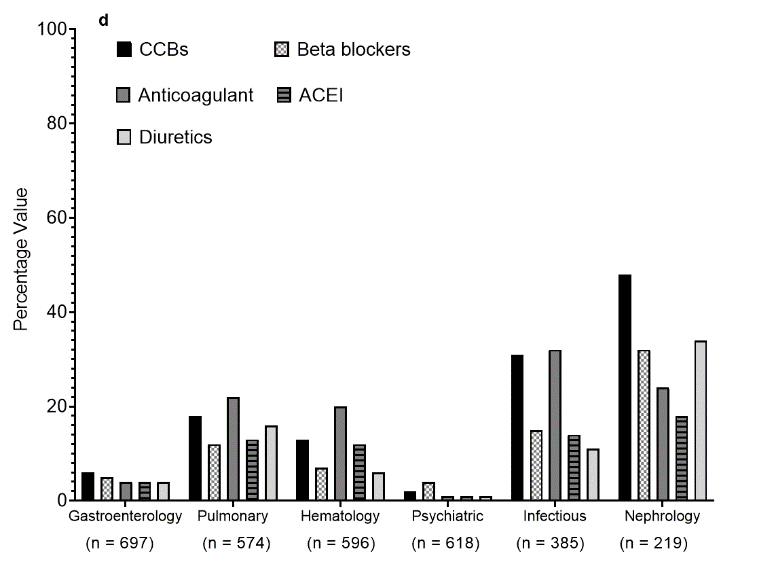 |

**Figure SM_3: Common recorded medications prescribing prevalence based on each clinic specialty. Data presents percentage value of the referenced group. NSAIDs = Non-Steroidal Anti-Inflammatory Drugs, CCBs = Calcium Channel Blockers, ACEIs = Angiotensin Converting Enzyme Inhibitors.**

Table SM_1: Common recorded morbidities among the cohort classified based on age group. GORD = Gastroesophageal reflux disease

| Comorbidity | Total Patients | Middle-aged Adults | Older Adults | *P*-value |
| --- | --- | --- | --- | --- |
|  | n = 14081 | n = 9722 | n = 4359 |  |
| Osteoarthritis, n (%) | 5262 (37) | 3793 (40) | 1469 (34) | < 0.001 |
| Ischemic heart disease, n (%) | 4783 (34) | 2543 (26) | 2240 (51) | < 0.001 |
| Osteoporosis, n (%) | 4616 (33) | 3225 (33) | 1391 (32) | 0.1 |
| Hypertension, n (%) | 4279 (30) | 2233 (23) | 2046 (47) | < 0.001 |
| GORD, n (%) | 4209 (30) | 2568 (26) | 1641 (38) | < 0.001 |
| Hyperlipidaemia, n (%) | 3738 (27) | 1877 (19) | 1861 (43) | < 0.001 |
| Anaemia, n (%) | 3726 (27) | 2649 (27) | 1077 (25) | 0.002 |
| Heart failure, n (%) | 3549 (25) | 1912 (19) | 1737 (40) | < 0.001 |
| Diabetes mellitus, n (%) | 2993 (21) | 1578 (16) | 1415 (33) | < 0.001 |
| Rheumatoid arthritis, n (%) | 2970 (21) | 2312 (24) | 658 (15) | < 0.001 |

Table SM_2: Prevalence of medications prescribing among the cohort classified based on the age groups. Medications were presented by the first level order as per the anatomical therapeutic classification.

| Medication Class | Total cohort | Middle aged adults | Older adults | *P*-value |
| --- | --- | --- | --- | --- |
|  | n = 14081 | n = 9722 | n = 4359 |  |
| Alimentary and metabolism, n (%) | 8043 (57) | 5150 (53) | 2893 (66) | < 0.001 |
| Musculo-skeletal system, n (%) | 6782 (48) | 4892 (50) | 1890 (43) | < 0.001 |
| Nervous system, n (%) | 6528 (46) | 4644 (48) | 1884 (43) | < 0.01 |
| Blood and blood forming organs, n (%) | 5943 (42) | 3687 (38) | 2256 (52) | < 0.001 |
| Cardiovascular system, n (%) | 5880 (42) | 3251 (33) | 2629 (60) | < 0.001 |
| Systemic hormonal preparation, n (%) | 2081 (15) | 1430 (15) | 651 (15) | 0.7 |
| Antineoplastic and immunomodulating agents, n (%) | 1506 (11) | 1307 (13) | 199 (5) | < 0.001 |
| Genito urinary system and sex hormones, n (%) | 1417 (10) | 684 (7) | 733 (17) | <0.001 |
| Respiratory system, n (%) | 1457 (10) | 892 (9) | 565 (13) | < 0.001 |
| Anti-infective for systemic use, n (%) | 1372 (10) | 916 (9) | 456 (11) | 0.06 |
| Sensory organs, n (%) | 643 (5) | 349 (4) | 294 (7) | < 0.001 |
| Dermatological, n (%) | 427 (3) | 253 (3) | 174 (4) | < 0.001 |
| Various, n (%) | 219 (2) | 108 (1) | 111 (3) | < 0.001 |
| Antiparasitic products, insecticides and repellents, n (%) | 127 (1) | 104 (1) | 23 (0.5) | 0.002 |

Table SM_3: Prevalence of medications prescribing among cohort classified based on the age groups. Medications were presented based on their therapeutics class. NSAIDs = non-steroidal anti-inflammatory drugs, ACEIs = Angiotensin converting enzyme inhibitors.

| Medication Class | Total cohort | Middle aged adults |  | Older adults | *P*-value |
| --- | --- | --- | --- | --- | --- |
|  | n = 14081 | n = 9722 |  | n = 4359 |  |
| Proton pump inhibitors, n (%) | 4209 (30) | 2568 (26) |  | 1641 (38) | < 0.001 |
| NSAIDs, n (%) | 4059 (30) | 3012 (31) |  | 1047 (24) | < 0.001 |
| Vitamin D, n (%) | 4178 (30) | 2985 (31) |  | 1193 (27) | < 0.001 |
| Statin, n (%) | 3711 (26) | 1856 (19) |  | 1855 (43) | < 0.001 |
| Antiplatelet, n (%) | 3018 (21) | 1423 (15) |  | 1595 (37) | < 0.001 |
| Calcium channel blockers, n (%) | 2358 (17) | 1181 (12) |  | 1177 (27) | < 0.001 |
| Beta blockers, n (%) | 2417 (17) | 1187 (12) |  | 1230 (28) | < 0.001 |
| Anticoagulants, n (%) | 1770 (13) | 971 (10) |  | 799 (18) | < 0.001 |
| ACEIs, n (%) | 1783 (13) | 977 (10) |  | 806 (19) | < 0.001 |
| Diuretics, n (%) | 1737 (12) | 729 (8) |  | 1008 (23) | < 0.001 |
